# Biochemical Modulators of Tight Junctions (TJs): Occludin, Claudin-2 and Zonulin as Biomarkers of Intestinal Barrier Leakage in the Diagnosis and Assessment of Inflammatory Bowel Disease Progression

**DOI:** 10.3390/molecules29194577

**Published:** 2024-09-26

**Authors:** Aleksandra Górecka, Agnieszka Jura-Półtorak, Ewa M. Koźma, Anna Szeremeta, Krystyna Olczyk, Katarzyna Komosińska-Vassev

**Affiliations:** Department of Clinical Chemistry and Laboratory Diagnostics, Faculty of Pharmaceutical Sciences in Sosnowiec, Medical University of Silesia in Katowice, 41-200 Sosnowiec, Poland; ajura@sum.edu.pl (A.J.-P.); mkozma@sum.edu.pl (E.M.K.); aszeremeta@sum.edu.pl (A.S.); olczyk@sum.edu.pl (K.O.); kvassev@sum.edu.pl (K.K.-V.)

**Keywords:** inflammatory bowel disease, Crohn’s disease, ulcerative colitis, occludin, claudin-2, zonulin, tight junctions, intestinal permeability

## Abstract

Background: Considering the increasing worldwide prevalence of inflammatory bowel disease (IBD), the early diagnosis of this disease is extremely important. However, non-invasive diagnostic methods remain limited, while invasive techniques are the most commonly used in daily practice. Therefore, there is a serious need to find new non-invasive biomarkers of IBD. Methods: The serum profiles of occludin, claudin-2, and zonulin were assessed in IBD patients using the ELISA method. The levels of the analyzed biomarkers were measured before and after a year of anti-inflammatory treatment, which was a tumor necrosis factor α (TNF-α) inhibitor (adalimumab) in patients with ulcerative colitis (UC) and conventional therapy in patients with Crohn’s disease (CD). Results: In IBD patients, the serum level of occludin (*p* < 0.001) decreased compared to healthy individuals, while the level of claudin-2 (*p* < 0.001) increased. Additionally, zonulin (*p* < 0.01) concentration increased in CD patients compared to the control group. The highest diagnostic ability was presented by occludin measurements with the area under the curve (AUC) of 0.959 (95% CI 0.907–1) in UC and 0.948 (95% CI 0.879–1) in CD. Claudin-2 also demonstrated very good ability in diagnosing UC and CD with AUC values of 0.864 (95% CI 0.776–0.952) and 0.896 (95% CI 0.792–0.999), respectively. The ability of zonulin to diagnose CD was estimated as good with an AUC of 0.74 (95% CI 0.598–0.881). Moreover, a significant correlation was identified between C-reactive protein (CRP), claudin-2 (r = −0.37; *p* < 0.05), and zonulin (r = −0.44; *p* < 0.05) in UC patients. Treatment with adalimumab improved the level of occludin, claudin-2, and zonulin in UC patients, while anti-inflammatory conventional therapy decreased the concentration of zonulin in CD. Conclusions: Occludin and claudin-2 measurements present significant utility in diagnosing both UC and CD, while zonulin assessments may be useful in CD diagnosis. Additionally, claudin-2 and zonulin measurements may be helpful in evaluating the intensity of the inflammatory process. Anti-TNF-α treatment improved the value of occludin, claudin-2, and zonulin, indicating its beneficial effect on the integrity of tight junctions in UC.

## 1. Introduction

Inflammatory bowel disease (IBD) comprises a group of inflammatory and chronic gastrointestinal disorders, among which ulcerative colitis (UC) and Crohn’s disease (CD) are the two main types. The pathogenesis of both UC and CD is complex, involving genetic predispositions, intestinal dysbiosis, disrupted intestinal barrier integrity, and excessive immune response. Although there are similarities in pathogenesis, the manifestation of UC and CD differs significantly. The inflammatory process in UC affects the colon in a continuous manner with characteristic erosions and ulcers, and it remains limited to the submucosa layer. In contrast, CD can affect any part of the gastrointestinal tract in a discontinuous pattern, with transmural inflammation, ulcers, strictures, fistulization, and granulomas. The course of both UC and CD is extremely burdensome for patients, involving alternating episodes of exacerbations and remissions [1,2,3,4,5]. Moreover, IBD is increasingly recognized as a significant global health issue, with its prevalence rising worldwide. The occurrence of IBD is particularly noted in Western countries, where the incidence is stabilizing, and in Eastern countries in Asia and Africa, where its incidence is rapidly growing. The high prevalence of IBD in Western countries combined with increasing incidence in Eastern countries makes IBD a global burden [6]. The early diagnosis of IBD is crucial to minimize the extent of the disease and prevent future complications. Currently, endoscopic examination is the “gold standard” for IBD diagnosis, though it involves a highly invasive examination. Among non-invasive diagnostic methods, fecal calprotectin is the most commonly used biomarker, indicating IBD with high sensitivity. However, fecal calprotectin is not specific to IBD, as it can be elevated in other gastrointestinal inflammatory diseases, and there is no consensus on its optimal cut-off, necessitating frequent retesting [7,8]. Therefore, there is a crucial need to identify new biomarkers that could support the early recognition of both UC and CD and reduce the reliance on invasive diagnostic methods.

Given the role of intestinal barrier integrity in the development of both UC and CD, biomarkers indicating barrier leakage may be useful for diagnosing and monitoring these diseases. The permeability of the intestinal barrier depends on tight junctions (TJs), which ensure the tightness of connections between intestinal epithelial cells (IECs) while allowing for the necessary paracellular flux. TJs are composed of transmembrane proteins, including occludin, junctional adhesion molecules, different types of claudins, and peripheral membrane proteins like zonula occludens. TJ permeability is dynamically regulated by various modulators, including TJ components like occludin [9,10]. Although the exact role of occludin in epithelial barrier function is not fully elucidated, numerous studies suggest that occludin plays a critical role in stabilizing TJs and maintaining barrier functions rather than TJ assembly. The downregulation of occludin is associated with increased permeability and chronic inflammation, indicating its possible role in IBD development [11,12]. Other proteins affecting TJ permeability include the claudin family, which comprises sealing claudins, which maintain TJ integrity, and pore-forming claudins (such as claudin-2), which enable paracellular transport. Inflammation in IBD often disrupts the balance between sealing and pore-forming claudins. Proinflammatory cytokines like tumor necrosis factor α (TNF-α) or interferon γ (IFN-γ) decrease the expression of sealing claudins while increasing the expression of pore-forming claudins, such as claudin-2. This upregulation during inflammation enhances the paracellular transport of Na^+^, water, and antigens, disrupting the intestinal barrier function [13,14]. Similarly, zonulin increases intestinal permeability activating protease-activated receptor-2 (PAR-2) and the epidermal growth factor receptor (EGFR), leading to the rearrangement of actin fibers and the dislocation of the zonula occludens 1. Considering that one of the triggers of zonulin release is bacterial presence, and intestinal dysbiosis contributes to IBD pathogenesis, the role of this protein in the development of the disease is further highlighted [15,16].

Considering the limited non-invasive diagnostic techniques and the necessity for an early IBD diagnosis, identifying novel sensitive and specific biomarkers is crucial. Given the role of intestinal barrier integrity in the development of IBD, the serum profiles of biochemical modulators of tight junctions, including occludin, claudin-2, and zonulin, may be useful in diagnosing both UC and CD. Moreover, these biomarkers may also be valuable in monitoring disease activity and the response to anti-inflammatory treatment in patients with UC and CD.

## 2. Results

### 2.1. Patients’ Characteristics

The characteristic of patients included in this research is presented in Table 1. In our study, 49 patients with IBD were further divided according to diagnosis into UC and CD groups. Among patients with UC, there were 12 females and 19 males. The median disease activity in the UC group was 3 points on the Mayo endoscopic scale; however, after a year of anti-inflammatory treatment with adalimumab, the disease activity score decreased to 2. The observed decrease in disease activity was noted as statistically significant (*p* < 0.001). Moreover, after the introduced therapy, the level of C-reactive protein (CRP) changed from 14.1 to 7.9 mg/L, which was also a statistically significant difference (*p* < 0.05). In this research, the serum profile of calprotectin was measured before and after biological treatment. Upon a year of biological therapy, the level of serum calprotectin decreased from 3337.1 to 2708.3 ng/mL; however, this difference was not statistically significant. The observed results indicate a beneficial effect of the implemented anti-TNF-α treatment in patients with ulcerative colitis. In the group of patients with Crohn’s disease, 8 females and 10 males were included. The mean disease activity before prednisone treatment was 303.4 points based on the Crohn’s disease activity index (CDAI) scale and 270.8 after therapy, a difference that was noted as statistically significant (*p* < 0.05). The mean CRP value also decreased from 20.6 to 15.8 mg/L; however, this change was noted as not statistically significant (*p* > 0.05). A similar observation was made in the case of serum calprotectin levels. Although the level of serum calprotectin was reduced after a year of anti-inflammatory treatment, this change was not significant (*p* > 0.05). Considering the notable difference in disease activity expressed on the CDAI scale, the provided conventional anti-inflammatory treatment was beneficial for patients with Crohn’s disease.

### 2.2. Differences in Occludin, Claudin-2, and Zonulin Serum Profiles between Patients with Inflammatory Bowel Disease and Healthy Individuals

Serum concentrations of occludin, claudin-2, and zonulin were measured in healthy individuals, and patients with UC and CD, and the results are presented in Table 2. According to the results from the obtained data (Table 2), the median level of occludin was equal in UC and CD patients, reaching the value of 0.35 ng/mL. A significant difference was, however, noted between the study and control groups; UC and CD patients presented a decreased level of occludin compared to healthy individuals (0.35 vs. 0.45 ng/mL; *p* < 0.001). In the case of claudin-2, the mean concentration was 2.72 ng/mL in the serum of UC patients, 3.17 ng/mL in CD patients, and 1.88 ng/mL in healthy individuals. Differences in the claudin-2 level were statistically significant between UC patients and healthy control (*p* < 0.001), as well as between CD subjects and the control group (*p* < 0.001). The last of the analyzed TJ proteins was zonulin, the mean concentration of which reached 21.92 in UC patients, 27.19 in CD patients, and 17.06 ng/mL in healthy individuals. The serum level of zonulin did not differ significantly between patients with UC and healthy individuals (*p* > 0.05); however, a significant increase in this parameter was noted in patients with CD compared to healthy individuals (*p* < 0.01). The serum profile of occludin, claudin-2, and zonulin was additionally compared between patients with ulcerative colitis and Crohn’s disease; however, no significant difference was noted between patients with these two types of IBD (*p* > 0.05).

### 2.3. ROC Analysis of Occludin, Claudin-2, and Zonulin Levels for Diagnosis of Ulcerative Colitis and Chron’s Disease

**Figure 1:**
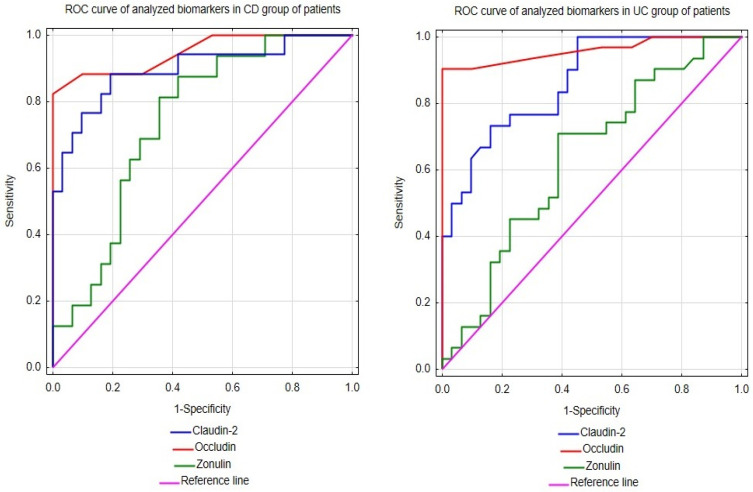
ROCs of occludin, claudin-2, and zonulin in patients with Crohn’s disease and ulcerative colitis.

In this study, to fully assess the utility of occludin, claudin-2, and zonulin measurements in the diagnosis of UC and CD, the receiver-operating characteristic curve (ROC) analysis was conducted for them, and the results are presented in Figure 1 and Table 3. The performed analysis of diagnostic usefulness revealed the excellent ability of the serum occludin concentration to differentiate UC from healthy individuals with an area under the curve (AUC) of 0.959 (Table 3). The optimal cut-off for the serum occludin level was established using the Youden index and determined as 0.45 ng/mL. At this cut-off, the circulating occludin had very good recognition of subjects with UC from people without the disease, as evidenced by the very high values of sensitivity and specificity, respectively (Table 3). Moreover, both the effectiveness and the power of positive or negative results of this parameter to diagnose or exclude UC, respectively, were very good based on high accuracy, positive predictive value (PPV), and negative predictive value (NPV) (Table 3). Similarly to the diagnosis of UC, the circulating occludin also had a high usefulness in CD patients, which was manifested by the excellent ability of this analyte to distinguish them from healthy people (AUC 0.948) (Table 3). In addition, this parameter was also characterized by high values of other indicators of diagnostic accuracy in CD patients (Table 3).

The ROC analysis for the serum concentration of occludin revealed that it had a very good ability to discriminate both patients with UC and CD from healthy subjects (AUC values of 0.864 and 0.896, respectively (Table 3)). At its optimal cut-off points, the serum concentration of claudin-2 was also characterized by high values of other diagnostic indicators in both patient groups (Table 3), although the diagnostic potential of this parameter seemed to be slightly higher in people with CD (Table 3).

The ROC analysis revealed that the serum concentration of claudin-2 showed only a sufficient ability to differentiate patients with UC or CD from healthy individuals (AUC values of 0.740 or 0.634, respectively (Table 3)). Moreover, at its optimal cut-off points, this biomarker had rather low values of other diagnostic indicators in both groups of patients, especially in people with UC (Table 3).

### 2.4. Usefulness of Occludin, Claudin-2, and Zonulin as Biomarkers Evaluating Disease Activity

In this research, we assessed the utility of occludin, claudin-2, and zonulin measurements in evaluating the disease activity among patients with ulcerative colitis and Crohn’s disease. In the group of patients with UC, no significant correlation was noted between the serum profile of the analyzed biomarkers and disease activity expressed on the Mayo scale. On the other hand, a significant relationship was noted between the serum level of CRP and claudin-2 (r = −0.37; *p* < 0.05), as well as between CRP and zonulin (r = −0.44; *p* < 0.05), but not between CRP and occludin *(p* > 0.05). In the group of patients with CD, none of the analyzed parameters correlated with either disease activity expressed on the CDAI scale (*p* > 0.05) or the CRP level (*p* > 0.05).

### 2.5. Influence of Anti-Inflammatory Treatment on the Serum Profile of Occludin, Cludin-2, and Zonulin in Patients with Ulcerative Colitis and Crohn’s Disease

**Figure 2:**
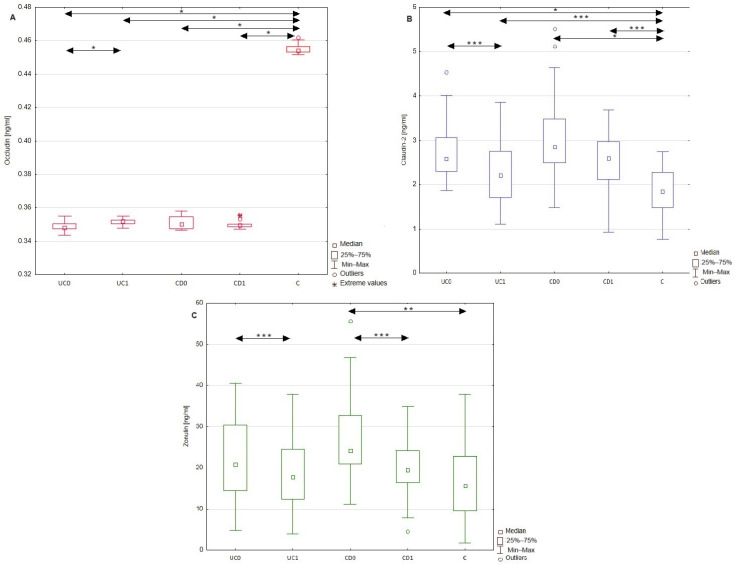
Comparison of serum occludin, claudin-2, and zonulin concentrations in healthy individuals, as well as patients with UC and CD. Serum profiles of occludin (**A**), claudin-2 (**B**), and zonulin (**C**) in the group of patients with ulcerative colitis and Crohn’s disease both before and after anti-inflammatory treatment, as well as in the control group. Data with statistical significance are marked with arrows. C, control group; CD0, patients with Crohn’s disease before treatment; CD1, patients with Crohn’s disease after treatment; UC0, patients with ulcerative colitis before treatment; UC1, patients with ulcerative colitis after treatment; *, *p* < 0.001; **, *p* < 0.01; ***, *p* < 0.05.

The serum profiles of occludin, claudin-2, and zonulin were assessed before and after a year of anti-inflammatory treatment in UC and CD groups of patients, whose data are graphically presented in Figure 2. Upon biological treatment with adalimumab in the UC group of patients, the serum level of occludin increased significantly by 0.002 ng/mL (*p* < 0.001). At the same time, the serum concentration of claudin-2 (*p* < 0.05) and zonulin (*p* < 0.05) decreased significantly, by 0.48 ng/mL and 3.07 ng/mL, respectively. Despite the mentioned changes after the implemented treatment, the serum profiles of occludin (*p* < 0.001) and claudin-2 (*p* < 0.05) were still significantly different from those of healthy individuals. In the group of patients with CD, no significant difference was noted in the serum levels of occludin and claudin-2 after a year of anti-inflammatory therapy. The post-treatment values of occludin (*p* < 0.05) and claudin-2 (*p* < 0.001) still differed between patients with CD and healthy individuals. The conducted statistical analysis revealed, however, a significant decrease in the zonulin concentration by 7.99 ng/mL in CD patients after therapy.

## 3. Discussion

### 3.1. Usefulness of Occludin, Claudin-2, and Zonulin as Diagnostic Markers in Ulcerative Colitis and Crohn’s Disease

The aim of this research was to identify new systemic biomarkers with potential use in the diagnostics of ulcerative colitis and/or Crohn’s disease. We focused on biochemical modulators of tight junctions, such as occludin, claudin-2, and zonulin. The results obtained revealed significant changes in circulating levels of most of these proteins in both patient groups compared to healthy controls. Therefore, the question arises whether these alterations may be related to the pathological processes that occur in the disease-affected intestine. We observed a statistically significant reduction in the level of circulating occludin in both UC and CD patients compared to healthy individuals. Interestingly, at least three distinct mechanisms in the pathologically altered intestine can lead to such a systemic manifestation. The first mechanism is impaired expression of occludin. The downregulation of this molecule at both the transcriptomic and protein levels was reported in biopsy samples from both inflamed and non-inflamed tissue areas, collected from patients with UC and CD [17]. Another mechanism that may be responsible for the reduction in the serum occludin concentration is related to the higher endocytosis of this protein in the disease-affected intestine. This process is under the control of several factors, including TNF α, which also plays a substantial role in the development of IBD. The detailed mechanism underlying this TNFα-promoted effect involves the activation of myosin light chain kinase, followed by actomyosin filament contraction and stimulation of the caveolin-1-dependent endocytosis of occludin [9,18,19]. Finally, the low level of circulating occludin in UC or CD patients might result from the extracellular degradation of this protein in the disease-affected intestine. The protein is a target for at least two matrix metalloproteinases (MMPs), namely MMP-2 and MMP-9, whose expression is increased in the colon of IBD patients [12,20].

In contrast to occludin, the serum concentration of claudin-2, another TJ component tested in this study, significantly increased in both UC and CD patients compared to the healthy controls. This phenomenon may reflect a strong upregulation of protein selectively in inflammation-affected areas of the intestine, which has been observed in both UC and CD patients [21]. The results of in vitro studies using cell culture models have shown that the expression of claudin-2, but not that of the other members of the claudin family, is stimulated by proinflammatory cytokines such as interleukin-6 (IL-6) or interleukin-13 (IL-13) [22,23], both of which are involved in the pathogenesis of IBD [14,22,23,24].

Furthermore, we found the level of circulating zonulin to be markedly elevated in CD patients compared to healthy subjects. Among patients with UC, the zonulin concentration also increased in comparison to healthy individuals; however, it was not as remarkable as in the CD group. Nevertheless, it cannot be ruled out that after increasing the number of CD patients, the differences in the circulating zonulin levels between these patients and the control group might become more apparent. In addition to our results, a significant increase in the circulating zonulin level in IBD patients compared to healthy individuals was also demonstrated by Kushlinskij et al. [25]. Interestingly, these authors found particularly high concentrations of the TJ protein in the blood of patients with colorectal cancer [25]. Moreover, the differences between the levels of circulating zonulin in cancer and IBD patients were statistically significant [25]. This finding may further suggest the utility of this parameter in the differential diagnosis of IBD and certain intestinal cancers. The elevated serum concentration of this TJ protein was also observed in IBD patients by Caviglia et al. [26]. The mechanism responsible for these alterations involves the upregulation of zonulin expression or its increased release from the proximal small intestine, but not from the colon, as a result of exposure to pathogenic or non-pathogenic microbial dysbiosis [15,27]. This effect on the local zonulin level is considered to be a host defense response to dysbiosis because this TJ modulator stimulates the paracellular flux, leading to the flushing out of bacteria [27]. Interestingly, only in CD does the pathological process affect any section of the gastrointestinal tract, including the small intestine, while in UC, lesions primarily involve the colon [1,2,27]. Thus, the induction of zonulin can be greater in CD patients than in UC ones.

In summary, the above-mentioned facts regarding alterations in the metabolism of occludin, claudin-2, and zonulin in the IBD-affected intestine suggest that the remodeling of the serum profiles of these proteins observed by us in UC and CD patients may actually be a systemic manifestation of these tissue processes. Therefore, the serum levels of the examined TJ components could have some potential in the diagnosis of IBD. Thus, we verified this hypothesis by performing the ROC analysis of the serum concentrations of these proteins in IBD patients and healthy people. The results obtained revealed that the serum profiles of occludin, claudin-2, and zonulin differed between patients with IBD and healthy individuals. To the best of our knowledge, this study is the first to identify that the circulating claudin-2, but especially occludin, had at least a very good ability to differentiate both UC and CD patients from healthy individuals. Moreover, both analytes were characterized by high values of the remaining indicators of the diagnostic usefulness. Therefore, the above results suggest that the serum concentrations of claudin-2 and occludin might be useful as non-invasive and sensitive markers in the preliminary diagnosis of both UC and CD.

In contrast to the circulating occludin or claudin-2, the serum concentration of zonulin had a poor ability to discriminate UC and CD patients from healthy people. However, considering the limited size of study groups, especially the CD group, the diagnostic utility of zonulin measurements in the serum might be improved if tested on a greater number of patients. However, despite the limited utility of zonulin for the diagnosis of IBD, the correlation between serum profiles of this parameter and CRP was significant in the UC group. Therefore, measurements of zonulin levels may be beneficial in monitoring and evaluating the inflammatory process during UC.

### 3.2. Influence of Anti-Inflammatory Treatment on the Serum Profile of Occludin, Claudin-2, and Zonulin in Patients with Ulcerative Colitis and Crohn’s Disease

In this study, the serum profile of occludin, claudin-2, and zonulin was also measured after a year of anti-inflammatory treatment with adalimumab in the group of patients with UC or with prednisolone in patients with CD. Both of these treatments led to a significant reduction in the disease activities. In the group of patients with UC, the implemented treatment significantly improved the value of all the analyzed biomarkers, increasing the concentration of occludin and decreasing the levels of claudin-2 and zonulin. However, it should be emphasized that among the examined TJ proteins, the observed alterations in the level of circulating occludin were the least intense in these patients. In turn, a year of anti-inflammatory therapy in CD patients significantly affected only the level of zonulin. Interestingly, similar to the UC group, in the CD patients, the level of circulating occludin demonstrated the highest resistance to the treatment. These results suggest that circulating zonulin might be a particularly sensitive marker of improvement in IBD patient status. In contrast, the metabolism of occludin seems to be especially sensitive to the presence of IBD even despite their treatment, further supporting the significant potential of this TJ protein to recognize these diseases. In addition, the values of circulating occludin in both IBD patients and healthy subjects demonstrated a narrow range of results, which may be related to low inter-individual variability within each of these groups, facilitating the practical use of the analyte.

The observed changes in occludin and claudin-2 levels in UC patients may be related to the amelioration of both disease activity and the inflammatory process, reflected by the CRP and serum calprotectin levels. Given the crucial role of proinflammatory cytokines on the expression of these two modulators of TJs, the diminished inflammatory process upon adalimumab treatment may be related to the observed improvement in occludin and claudin-2 levels in UC patients. Considering that adalimumab is a biological inhibitor of TNF-α, the introduced therapy may inhibit the TNF-α-mediated downregulation of occludin. This hypothesis is supported by the results obtained by Xu et al. [28]. In that study, researchers evaluated the influence of plasma collected from patients with CD on TJs in a Caco-2 cell model. The exposure of Caco-2 cells to CD plasma increased the permeability of TJs by decreasing the mRNA and protein levels of occludin. Similar results were obtained upon exposure of Caco-2 cells to TNF-α, indicating the prominent role of this cytokine in the impairment of TJs. At the same time, the pre-incubation of Caco-2 cells with adalimumab prevented the disruption of barrier integrity. These results, together with the improvement in occludin levels upon adalimumab treatment, may indicate the beneficial role of TNF-α inhibitors in the integrity of TJs.

Moreover, in this study, one year of anti-TNF-α treatment affected the concentration of another TJ protein claudin-2 in patients with UC. TNF-α is engaged in the activation of the inflammatory response via the nuclear factor κB pathway, whose activation is followed by the synthesis and release of proinflammatory cytokines, including TNF-α, IL-1, and IL-6. The administration of TNF-α inhibitors decreases the TNF-α-mediated release of proinflammatory cytokines, regulating the expression of TJ proteins such as claudin-2 [29]. The studies by Dahlén et al. [30] and Ringheanu et al. [31] revealed that the incubation of immune cells isolated from IBD patients with anti-TNF-α antibodies (infliximab) reduced the expression and secretion of proinflammatory cytokines, including IL-6 and IL-13. Therefore, the administration of adalimumab may decrease the release of IL-6 and IL-13 from immune cells during UC, thereby decreasing the levels of these cytokines, which enhance claudin-2 expression. To our knowledge, this study is the first to indicate the effect of adalimumab treatment on the serum profiles of occludin and claudin-2 in patients with UC. In patients with CD, no significant difference in either occludin or claudin-2 levels was observed after treatment with prednisolone. These observations may indicate that anti-inflammatory treatment with glucocorticosteroids does not improve the expression of at least some TJ components. This suggestion should, however, be further analyzed since studies on the influence of glucocorticosteroids on TJ proteins in IBD patients remain limited.

The only biochemical modulator of TJs (almost) normalized after one year of anti-inflammatory treatment in both groups of IBD patients, undergoing both anti-TNF-α and glucocorticosteroid treatments, was zonulin. Considering that the triggers of zonulin expression are not proinflammatory cytokines but intestinal microbiota, the implemented treatment may not affect the zonulin level directly. The observed decrease in zonulin levels in both UC and CD patients may be the result of the beneficial effect of the implemented treatments on the permeability of tight junctions between intestinal epithelial cells. In fact, in the study conducted by Wegh et al. [32], serum zonulin levels correlated with intestinal permeability assessed using the sucralose test in patients with UC. Therefore, zonulin measurements in the serum may serve as a marker of intestinal permeability, which at the same time is not dependent on the influence of proinflammatory cytokines. The observed decrease in zonulin serum profiles in patients with UC and CD may indicate a decrease in intestinal permeability, but this conclusion should be verified with other validated intestinal permeability tests.

This study has some limitations, among which the size of the study groups should be mentioned, especially in the case of CD patients. The limited number of patients was due to including only newly diagnosed patients and strict inclusion criteria for participation in this study. Increasing the number of patients would provide a more accurate estimation of the usefulness of the examined tight junction proteins in clinical practice for IBD. Moreover, this assessment could be further improved by introducing a second control group, including patients with gastrointestinal diseases other than IBD, into the study. The reliability of the analyzed TJ protein as biomarkers of IBD should also be confirmed using a cellular IBD model to further characterize the role of occludin, claudin-2, and zonulin in the development of the disease. On the other hand, our study demonstrates for the first time the great potential usefulness of the circulating TJ proteins for the non-invasive diagnostics of IBD. In addition, these possible markers can be measured using simple and validated tests, which enables their quick implementation into common laboratory practice. Another strength of our study is the inclusion of patients with both types of IBD, i.e., UC and CD, which makes it possible to identify potential differences between them with regard to the mechanism(s) of the intestinal barrier damage. Moreover, since UC and CD patients were introduced to different treatments—biological TNF-α inhibitors vs. anti-inflammatory corticosteroids—this research also assessed the influence of these two kinds of therapy on the level of circulating TJ proteins. The obtained results may therefore contribute to the development of future effective strategies, at least in the diagnostics of IBD.

## 4. Materials and Methods

### 4.1. Study Population

The study group for this research included 31 patients with ulcerative colitis and 18 patients with Crohn’s disease. The inclusion criteria for participation in this research were active and newly diagnosed disease, as well as age above 18 years old. The exclusion criteria included unstable coronary disease; toxic or fulminant colitis; severe bacterial, fungal, or viral infections; chronic liver or kidney diseases; pregnancy; and breastfeeding. The diagnosis of the diseases was made on the basis of clinical symptoms, laboratory tests, and endoscopic examination at the Department of Gastroenterology of St. Barbara’s Regional Specialist Hospital in Sosnowiec. The activity of the disease was also evaluated at the St Barbara’s Hospital in Sosnowiec and was based on the Mayo endoscopic scale in the case of ulcerative colitis and the CDAI in Crohn’s disease patients. The biological material investigated in this research was venous blood collected from patients with UC and CD before and after a year of treatment. Patients with ulcerative colitis received the biological anti-TNF-α treatment (adalimumab), while the treatment of patients with Crohn’s disease included anti-inflammatory corticosteroids (prednisolone). In the group of patients with Crohn’s disease, the prednisolone doses were determined on a case-by-case basis depending on the activity of the disease. After the amelioration of the acute flare, the doses were reduced gradually over 2–3 months until complete discontinuation.

The control group for this research included 31 healthy individuals. The inclusion criteria for participation in this research were age above 18 years old, no surgery 12 months prior to the study, and no current pharmacological treatment. Moreover, the results of routinely assessed hematological and biochemical parameters had to be within the reference range for their particular sex and age group. The material collected from healthy individuals was venous blood.

### 4.2. Measurements of Tight Junction Proteins (Occludin, Claudin-2, and Zonulin) by ELISA

The concentration of occludin, claudin-2, and zonulin was assessed in the serum of healthy individuals, as well as patients with UC and CD. The serum profile of occludin was measured using an ELISA test for occludin from Cloud-Clone Corporation (Houston, TX, USA) (catalog no.: SEC228Hu). The intra-assay precision of this test was <10%, while the sensitivity was 0.059 ng/mL. In this test, the detection limit was 0.156 ng/mL. The concentration of claudin-2 was analyzed with an ELISA test for claudin-2 from Cloud-Clone Corporation (Houston, TX, USA) (catalog no.: SEF292Hu), which was characterized by an intra-assay precision <10% and sensitivity equaling 0.058 ng/mL. Moreover, the detection limit of this test was 0.156 ng/mL. Zonulin levels in the serum of healthy individuals and IBD patients were assessed using the zonulin ELISA test from Immunodiagnostik AG Company (Berlin, Germany) (catalog no.: K 5601). The antibodies used in this test were polyclonal and directed toward zonulin sequences published in the studies by Wang et al. [33] and di Pierro et al. [34]. The intra-assay precision of this test was estimated at 4.75%, while the sensitivity was 0.183 ng/mL. The detection limit of this test was 0.183 ng/mL.

### 4.3. Statistical Analysis

Statistical analysis for this research was conducted using the STATISTICA software (version 13.3) from StatSoft (Cracov, Poland). The performed analysis included the Shapiro–Wilk test to estimate the normality of the data distribution, Student’s *t*-test, and the Mann–Whitney U test to assess the significance of differences in the analyzed parameters between the groups, as well as before and after therapy in the study groups. Data with a normal distribution are presented in this manuscript as mean ± standard deviation, while non-normally distributed data are presented as median with interquartile range in Table 1 and Table 2. In the case of normally distributed data, Student’s *t*-test was used, while non-normally distributed data were analyzed with the Mann–Whitney U test. Additionally, the analysis of the ROC was conducted to evaluate the diagnostic utility of the analyzed biomarkers. The correlation of the assessed parameters with disease activity and inflammatory biomarkers (CRP) was estimated based on the Pearson test (normally distributed data) and the Spearman test (non-normally distributed data). For all statistical analyses, the significance level of *p* < 0.05 was used.

## 5. Conclusions

The aim of our study was to identify new parameters that could support the diagnosis of both ulcerative colitis and Crohn’s disease. Among the tested analytes, the most promising biomarkers were occludin and claudin-2, which demonstrated (displayed) a very good ability to diagnose both UC and CD. Furthermore, given the relationships between claudin-2, zonulin, and CRP in UC, these biochemical modulators of TJs could also reflect the intensity of the inflammatory process. Moreover, the obtained results indicate the beneficial influence of TNF-α inhibitors on the intestinal barrier, as upon treatment, the concentration of all biochemical modulators of TJs improved among patients with UC.

## Figures and Tables

**Table 1:** Clinical characteristics of patients included in this study.

	Patients with Ulcerative Colitis			Patients with Crohn’s Disease		
	Before Treatment UC0	After Treatment UC1	*p* UC0 vs. UC1	Before Treatment CD0	After Treatment CD1	*p* CD0 vs. CD1
Number of patients	31		n.a.	18		n.a.
Sex [females/males]	12/19		n.a.	8/10		n.a.
Age [years]	33.4 ± 12.8		n.a.	32.1 ± 9.6		n.a.
BMI [kg/m^2^]	24.3 ± 3.6	24.5 ± 4.2	>0.05	20.6 ± 3.4	19.8 ± 2.8	>0.05
Mayo score	3 (2–3)	2 (1–3)	**<0.001**	n.a.	n.a.	n.a.
CDAI score	n.a.	n.a.	n.a.	303.4 ± 52.5	270.8 ± 44.3	**<0.05**
CRP [mg/L]	14.1 ± 24.1	7.9 ± 13.3	**<0.05**	20.9 ± 21.1	15.8 ± 11.4	>0.05
Serum calprotectin [ng/mL]	3337.1 ± 1775.3	2708.3 ± 890.9	>0.05	3537.5 ± 1893.8	2915.3 ± 1325.9	>0.05
Albumin [g/L]	42 (40–46)	43 (40–48)	>0.05	43.5 (42–47.3)	43.5 (42–49)	>0.05
Creatinine [μmol/L]	77.8 (68.5–87.9)	74.7 (63.4–87.1)	>0.05	81.3 ± 14.1	86.6 ± 13.2	>0.05
Glucose [mmol/L]	4.9 ± 0.7	4.8 ± 0.8	>0.05	5.1 ± 0.9	4.9 ± 0.4	>0.05
AST [U/L]	19.0 (14–46)	19 (15–23)	>0.05	21.5 (18.5–24.3)	21 (16.8–23.5)	>0.05
ALT [U/L]	15 (10–26)	16 (10–25)	>0.05	24 (16.3–29)	21.5 (14.5–31)	>0.05
Na [mmol/L]	140 (138–142)	140.00 (138–141)	>0.05	138.2 ± 2.9	138.4 ± 3.9	>0.05
K [mmol/L]	4.2 ± 0.4	3.9 ± 0.3	**<0.05**	4.4 (4.2–4.5)	4.4 (4.2–4.5)	>0.05
Hemoglobin [g/dL]	12.8 ± 2.3	13.5 ± 2.3	<0.01	11.6 ± 2.3	12.4 ± 1.9	>0.05
White blood cell count [×10^3^/μL]	7.9 (3.9–13.7)	7.1 ± 3.2	>0.05	6.7 ± 2.1	6.7 ± 2.1	>0.05
Platelet count [×10^9^/L]	375.9 ± 108.8	342 ± 101.7	**<0.05**	356.5 (277.5–396)	232.2 (134.2–309.1)	>0.05

Comparison of selected hematological and biochemical parameters assessed in patients with ulcerative colitis and Crohn’s disease before and after treatment. Data are presented as mean ± standard deviation in normally distributed data and median with interquartile range in non-normally distributed data. Data with statistical significance are shown in bold. ALT, alanine aminotransferase; AST, aspartate aminotransferase; BMI, body mass index; CD0, patients with Crohn’s disease before treatment; CD1, patients with Crohn’s disease after treatment; CDAI, Crohn’s disease activity index; CRP, C-reactive protein; K, potassium; Na, sodium; n.a., not applicable; UC0, patients with ulcerative colitis before treatment; UC1, patients with ulcerative colitis after treatment.

**Table 2:** Serum profile of occludin, claudin-2, and zonulin in patients with ulcerative colitis and Crohn’s disease, and healthy individuals.

Parameter	UC0	UC1	*p* UC0 vs. UC1	CD0	CD1	*p* CD0 vs. CD1	C	*p* UC0 vs. C	*p* CD0 vs. C
Occludin [ng/mL]	0.349 ± 0.003	0.351 ± 0.002	<0.001	0.353 (0.349–0.357)	0.350 (0.349–0.353)	>0.05	0.454 (0.453–0.457)	<0.001	<0.001
Claudin-2 [ng/mL]	2.72 ± 0.66	2.24 ± 0.76	<0.05	3.17 ± 1.12	2.51 ± 0.75	>0.05	1.88 ± 0.48	<0.001	<0.001
Zonulin [ng/mL]	21.92 ± 9.29	18.85 ± 8.04	<0.05	27.19 ± 11.38	19.2 ± 7.65	<0.05	17.06 ± 10.15	>0.05	<0.01

Results are presented as mean ± standard deviation in normally distributed data and median with interquartile range in non-normally distributed data. C, healthy control; CD0, patients with Crohn’s disease before treatment; CD1, patients with Crohn’s disease after treatment; UC0, patients with ulcerative colitis before treatment; UC1, patients with ulcerative colitis after treatment.

**Table 3:** The results of ROC analysis of the biomarkers.

Patients	Parameter	AUC	Cut-Off	Youden Index	Accuracy	Sensitivity	Specificity	PPV	NPV
UC	Occludin	0.959 (95% CI 0.907–1)	0.45 ng/mL	0.90	95%	90%	100%	100%	91%
	Claudin-2	0.864 (95% CI 0.776–0.952)	2.33 ng/mL	0.57	79%	73%	84%	79%	76%
	Zonulin	0.634 (95% CI 0.495–0.774)	17.52 ng/mL	0.32	66%	71%	61%	65%	68%
CD	Occludin	0.948 (95% CI 0.879–1)	0.36 ng/mL	0.82	93%	88%	90%	100%	91%
	Claudin-2	0.896 (95% CI 0.792–0.999)	2.49 ng/mL	0.69	85%	77%	90%	81%	88%
	Zonulin	0.740 (95% CI 0.598–0.881)	20.78 ng/mL	0.46	70%	81%	65%	54%	87%

AUC, the area under the ROC; CD, Crohn’s disease; NPV, negative predictive value; PPV, positive predictive value; UC, ulcerative colitis.

## Data Availability

Data are contained within the article.

## Abbreviations

ALT	alanine aminotransferase
AUC	area under curve
AST	aspartate aminotransferase
BMI	body mass index
CRP	c-reactive protein
CD0	patients with Crohn’s disease before treatment
CD1	patients with Crohn’s disease after treatment
CDAI	Crohn’s disease activity index
EGFR	epidermal growth factor receptor
IBD	inflammatory bowel disease
IFN-γ	interferon-γ
IL-6	interleukin-6
IL-23	interleukin-23
IECs	intestinal epithelial cells
MMPs	metalloproteinases
Na	sodium
NPV	negative predictive value
PPV	positive predictive value
K	potassium
PAR	protease-activated receptor
ROC	receiver-operating characteristic
TJs	tight junctions
TNF-α	tumor necrosis factor α
UC0	patients with ulcerative colitis before treatment
UC1	patients with ulcerative colitis after treatment
